# Measuring Health Utilities in Children and Adolescents: A Systematic Review of the Literature

**DOI:** 10.1371/journal.pone.0135672

**Published:** 2015-08-14

**Authors:** Dominic Thorrington, Ken Eames

**Affiliations:** Centre for the Mathematical Modelling of Infectious Diseases, London School of Hygiene and Tropical Medicine, London, United Kingdom; University of Exeter, UNITED KINGDOM

## Abstract

**Background:**

The objective of this review was to evaluate the use of all direct and indirect methods used to estimate health utilities in both children and adolescents. Utilities measured pre- and post-intervention are combined with the time over which health states are experienced to calculate quality-adjusted life years (QALYs). Cost-utility analyses (CUAs) estimate the cost-effectiveness of health technologies based on their costs and benefits using QALYs as a measure of benefit. The accurate measurement of QALYs is dependent on using appropriate methods to elicit health utilities.

**Objective:**

We sought studies that measured health utilities directly from patients or their proxies. We did not exclude those studies that also included adults in the analysis, but excluded those studies focused only on adults.

**Methods and Findings:**

We evaluated 90 studies from a total of 1,780 selected from the databases. 47 (52%) studies were CUAs incorporated into randomised clinical trials; 23 (26%) were health-state utility assessments; 8 (9%) validated methods and 12 (13%) compared existing or new methods. 22 unique direct or indirect calculation methods were used a total of 137 times. Direct calculation through standard gamble, time trade-off and visual analogue scale was used 32 times. The EuroQol EQ-5D was the most frequently-used single method, selected for 41 studies. 15 of the methods used were generic methods and the remaining 7 were disease-specific. 48 of the 90 studies (53%) used some form of proxy, with 26 (29%) using proxies exclusively to estimate health utilities.

**Conclusions:**

Several child- and adolescent-specific methods are still being developed and validated, leaving many studies using methods that have not been designed or validated for use in children or adolescents. Several studies failed to justify using proxy respondents rather than administering the methods directly to the patients. Only two studies examined missing responses to the methods administered with respect to the patients’ ages.

## Introduction

### Rationale

Evaluation of healthcare interventions and technologies commonly assess both the cost and consequences of interventions, in addition to effectiveness and safety. Economic evaluations are increasingly being used by healthcare systems around the world before a decision is made on whether to recommend a new intervention. In the United Kingdom, for example, the National Institute for Health and Care Excellence (NICE) requires that the appraisal of new interventions and technologies includes a cost-effectiveness analysis containing an assessment of benefits and resource use [1]. A requirement in the evidence submitted is a cost-utility analysis (CUA) that compares costs with benefits using quality-adjusted life years (QALYs), a measure incorporating the length of life and quality of life.

Quality of life is measured using health utilities that take values between 0 and 1, corresponding to utilities for dead and perfect health respectively. These utilities measured pre- and post-intervention are combined with the time over which the health states are experienced to calculate the QALYs that can be gained from new interventions. When evaluating several new health technologies the ratio of expected additional total costs to the expected additional QALYs gained incrementally is estimated for each technology, then cost-effectiveness is evaluated by comparing the incremental cost-per-QALY ratio against a pre-determined cost-effectiveness threshold, which in the UK is between £20,000 and £30,000 per QALY gained [1].

A CUA is also the recommended economic evaluation for submissions to the Canadian Agency for Drugs and Technologies in Health (CADTH) [2]; in Australia with submissions to The Pharmaceutical Benefits Advisory Committee (PBAC) [3]; in Sweden with submission to The Swedish Council on Health Technology Assessment (SBU) [4]; in New Zealand with submissions to The Pharmaceutical Management Agency (PHARMAC) [5] and other countries [6].

Health state utility values are usually obtained from one of two sources. Either the relevant health states are directly valued, using techniques such as Time Trade Off (TTO) or Standard Gamble (SG), or an existing tariff is applied. This latter approach is generally used when valuing generic health states (such as the EuroQol EQ-5D [7]). The tariff to be applied is usually based on valuations of a general population sample again using techniques such as TTO and SG. The TTO is a choice-based method that establishes for an individual how much time in full health is equivalent to a specified period of time spent in a particular ill-health state. The SG is another choice-based method that identifies the probability of being in a better health state that makes an individual indifferent between the certainty of being in an intermediate health and a gamble between a worse health state and a better health state.

Measuring utilities for health-related quality of life (HRQoL) for children and adolescents is a developing field of research. Methods used to obtain health utilities from adults are well established but many have not been validated for use in children and adolescents. NICE states that the EQ-5D is the preferred method for use in CUAs that focus on the adult population [1], but no specific guidance has been given to help health economists choose an instrument designed for children and adolescents. Indeed, NICE did not make a specific recommendation for a particular instrument in the publication of their most recent guidance on technology appraisal [1].

There is evidence that children and adolescents are able to report on the state of their own health [8]. Children aged 3 years can report on feelings of nausea and pain that are reliable and clinically meaningful [9–11]. If children can convey the state of their health using a standardised method such as EQ-5D or HUI-2 then accurate and meaningful health utilities may be obtained for a range of childhood illnesses and conditions, which would be highly desirable for conducting CUAs.

It is important to recognise that methods suitable for young children may not be applicable to adolescents [12, 13], in the same way that adult-specific methods may not be appropriate for recording health utilities of adolescents [14]. Children may lack the cognitive ability to evaluate their health using abstract concepts in adult-specific indirect methods and direct methods such as TTO and SG. In addition, young children may lack the required linguistic skills to answer questions about their preferences for health using systems designed for self-completion by older children. The understanding of disease and its effect on HRQoL changes with the child’s age, consequently both the measurement and valuation of changes in health due to disease need to be facilitated using age-specific instruments [12, 15].

Some methods have been developed for use exclusively in children and adolescents, and some existing adult-specific methods have been modified to make them child-friendly. The EQ-5D has been amended so that the questions for each dimension of health are easier to read and more accessible to children, resulting in a new child-friendly method called the EQ-5D-Y [16]. However, this uses the same utility weights in each dimension as the adult version, so does not yet incorporate child and adolescent preferences for health states. Adult preferences for health states may be different from the preferences of children and adolescents and the dimensions included may not cover all dimensions of health relevant to children and adolescents [17].

#### Generic and disease-specific calculation methods

Direct and indirect methods for the calculation of health utilities fall into two distinct domains–generic and disease-specific. Generic methods can be used to measure HRQoL in adults, children and adolescents (where appropriate) for a range of conditions, both chronic and acute. Commonly used generic methods include the EQ-5D and HUI-2. Disease-specific methods measure HRQoL with reference to a particular condition, such as the Asthma Control Questionnaire (ACQ) [18] and the Pediatric Asthma Health Outcome Measure (PAHOM) [19].

The advantage of using generic calculation methods in CUAs is that results can be compared across populations, conditions, and for different treatments or interventions [20]. Disease-specific methods have the benefit of being more sensitive to small changes in the condition of the patient in question and may describe the functioning of a patient with the condition with greater clarity than a generic classification system that may overlook some aspects of HRQoL [21], but utilities calculated using these instruments lack comparability across different diseases.

#### Measurement by proxy

When measuring the HRQoL of young children some authors prefer to gather the health utilities via proxies as young children may not have the cognitive ability to evaluate their health and/or complete the required measurement tasks [17]. Proxy respondents include the child’s parents, clinicians and teachers. Parents are deemed to be the most useful proxies as they are the most familiar with their child’s health and life [22, 23], though it has been suggested that parents may misjudge the health of their child owing to their own anxiety during the illness [24, 25] and further studies have shown differences between parent and child ratings for the child’s health [26–28]. Clinicians’ knowledge of children’s conditions, symptoms, and functioning makes them useful proxies when evaluating HRQoL, though they will not have the same contact with children during their time away from clinics at home or in school [22, 29] so results are of questionable validity [30]. Teachers will not be able to provide HRQoL assessments for the child at home or in clinics [22] but will be able to evaluate a child’s emotional and physical functioning.

In a systematic review published in 2005, Griebsch et al. [31] concluded that methods for measuring health utilities in children need further development. They noted the lack of methods that account for the development of the child, methods for children aged younger than 5 years, and a full understanding of the role of proxies in the evaluation of HRQoL in children and adolescents. Ravens-Sieberer et al. (2006) concluded that HRQoL of children and adolescents can and therefore should be ascertained by self-rating [32].

When performing a CUA in children and adolescents researchers must determine the best way to obtain utilities: expert opinion, measurement using patients or measurement using proxies. Each option will impose limitations on the study, and if the protocol calls for measurement then the researchers need to choose the appropriate method. The method used in CUAs should be justified as each has limitations relevant to the estimation of health utilities and QALYs.

### Objective

The objective of this review was to evaluate the application of direct and indirect methods used to measure health-related quality of life in children and adolescents. In doing so, we aimed to answer the following questions:

1. What direct and indirect methods have been used to obtain health utilities from children and adolescents? How frequently have they been used?
2. If the method has not been validated for use in the study population do the authors acknowledge the limits of the method and therefore the study?
3. For study populations that include adults with children and adolescents, did the younger participants complete the calculation method to the same level as the adult participants?
4. When proxies have been used to obtain health utilities have the authors acknowledged the problems related to obtaining such utilities from proxies rather than patients?

### Previous reviews

Kromm et al. (2012) [14] used the Pediatric Economic Database Evaluation (PEDE) project’s online database to find a total of 213 CUAs for children and adolescents published in English between 1997 and 2009 to use in a quality appraisal. Citing that CUAs were 8% of all published economic evaluations between 1976 and 2001 [33] and also that 10% of economic evaluations for children and adolescents published between 1980 and 1999 were CUAs [34], they assessed the quality of such CUAs using the 57-item Pediatric Quality Appraisal Questionnaire (PQAQ) [35]. Only 16 (8%) of the studies included in the review gathered health utilities as part of the analysis (Table 1).

**Table 1:** Results from Kromm et al. (2012) [14] for studies that measured health utilities as part of the CUA.

Were health utilities measured in the study?	From whom?	Direct measurement methods used	Indirect measurement methods used
Yes (n = 16)	Child (n = 5)	Time trade-off	EuroQol EQ-5D
	Parent as proxy (n = 10)	Standard Gamble, Time trade-off, Visual Analogue Scale	EuroQol EQ-5D, Health Utilities Index, Quality of Well-Being Scale
	Health care provider as proxy (n = 3)	None	EuroQol EQ-5D, Health Utilities Index, 16D-questionnaire
	Adults as proxy (n = 1)	Time trade-off	None
	Parent as unit of analysis (n = 1)	Time trade-off	None

Other studies used health utilities from the researchers or literature (63%), health care provider opinion (6%), disability-adjusted life years (DALYs) (25%) and the remainder did not state the source of the utilities (1%). Kromm et al. (2012) argued that utilities gathered from the published literature might not be valid [36]. Study authors may assume that adult health utilities apply to children and adolescents and assume a uniform utility throughout childhood and adolescence, ignoring the child’s development [12, 13]. In conclusion, the authors stated that new instruments should be developed to obtain utilities from children, rather than relying on adult utilities from the literature and utilities gathered via proxy.

Ladapo et al. (2007) [37] concentrated on CUAs in the United States, comparing analyses for adult, children and adolescent interventions. Using a database developed by the Tufts-New England Medical Center in Boston, they compared various aspects of 35 CUAs for children and adolescents with 491 adult CUAs. They found that generic classification systems (EQ-5D, Quality of Well Being (QWB) and HUI only) were used in 29% of analyses for children and adolescents and such CUAs are methodologically similar to adult CUAs. The leading primary disease category for CUAs for children and adolescents was infectious, representing 31% of all such CUAs. Finally, the authors noted that published cost-utility ratios tend to be lower for children and adolescents than for adults.

Griebsch et al. (2005) [31] considered all CUAs for patients aged younger than 17 years published until April 2004 in the Medline, Embase, Econlit, York Database of Abstracts of Reviews of Effectiveness, NHS Economic Evaluation Database, the Harvard Cost-Utility Analysis Database and the Database of the PEDE project. 63 direct or indirect calculation methods were used to estimate health utilities, of which 22 (35%) used a generic method. The authors concluded that the variation in methods for estimating health utilities in children and adolescents meant that the process was not yet standardised. They called for the clear justification of the choice of methods for measurement.

Recently, Adlard et al. (2014) [38] discussed how the practice of paediatric CUAs has evolved over time, with reference to methods described in the NICE reference case [1]. The review considered 43 studies published between May 2004 and April 2012, of which only 11 obtained health utilities from children with the remaining 32 studies using utilities published in the literature. The authors noted that since NICE suggested investigators use the HUI-2 to obtain health utilities from children there has been no increase in use of this instrument, with many authors seeking to use the EuroQol EQ-5D or its derivatives. Adlard et al. recommended that research funding be targeted at those studies seeking to estimate health utilities directly from children, given a lack of published data specific to this age group and wide variation in the methods used to obtain these data in previous work.

In contrast to the reviews cited, this review examined the methods used by researchers and health economists to estimate health utilities for children and adolescents and the extent of the variation between them. Details of all methods administered in each study were collated to evaluate the suitability of each system given the age of study participants, mode of completion and the stated justification for use of each calculation method.

## Methods

### Eligibility criteria

Studies eligible for inclusion in the final review needed to include primary data to measure health utilities from patients aged 17 years or under, through the administration of at least one direct or indirect method completed by either the patients or their proxies. Studies that included adult patients were not excluded, but studies that gathered HRQoL data exclusively from adults were excluded. We did not exclude studies based on language of publication, date of publication, journal or disease.

Studies that used other methods to calculate HRQoL scores that are incapable of generating utilities without a further mapping process were excluded unless the study also used a method to calculate health utilities.

Eligibility was not restricted to CUAs using primary data for HRQoL; studies detailing the validation of methods and studies that calculated health utilities for specified conditions but stopped short of collecting data related to healthcare resource use and patient-borne costs to calculate a cost-per-QALY ratio were eligible for inclusion.

Studies using health utilities gathered from previous studies were excluded, as were reviews, comment pieces and conference abstracts. All studies included in the full-text review had their references checked for additional studies to include in the review that did were not found through the online database search.

### Information sources

We searched for articles in the following databases: CAB Abstracts, Global Health, Ovid MEDLINE(R), Econlit and Embase Classic+Embase.

### Search

The search terms were taken from a systematic review published in 2005 by Griebsch et al. [31], appraising published CUAs in child and adolescent health care and looking at further issues still in doubt within the measurement of HRQoL in children and adolescents:

1. Infant, newborn/
2. Infant/
3. Child, preschool/
4. Child/
5. Adolescent/
6. 1 or 2 or 3 or 4 or 5
7. *expand* quality-adjusted life years/
8. cost-utility or cost utility
9. cost-effectiveness or cost effectiveness
10. 7 and 9
11. 8 or 10
12. 11 and 6

The search was performed on 30^th^ September 2014.

### Data items

The following data were extracted from papers included in the full-text review:

- Reference
- Year of publication
- Country
- Direct or indirect calculation method(s) used
- Health condition (if applicable)
- Sample size
- Age range of participants
- Mode of assessment:
  ○ Self-completion of questions
  ○ Completion of questions via proxy (parents, clinicians, primary caregivers, etc.)
  ○ Patient interviews
  ○ Interviews with proxies (parents, clinicians, primary caregivers, etc.)
  ○ Other methods
  ○ Methods not stated
- Study type:
  ○ Validation of calculation method
  ○ CUA
  ○ Health utility assessment
  ○ Comparison of calculation methods

We classified each study as one of four study types by the primary aim of each study: validations of calculation methods sought to validate or derive an instrument for estimating health utilities; CUAs first estimated health utilities then used these utilities in an economic evaluation; health utility assessments measured the burden of disease in individuals using health utilities; and comparisons of calculation methods used two or more instruments to measure health utilities then compared results.

In addition, each paper was analysed to ascertain whether or not the method(s) used had been justified for use in the cohort, along with the acknowledgment of any data collection issues that were related to the participants’ understanding of the calculation method.

### Results

**Fig 1:**
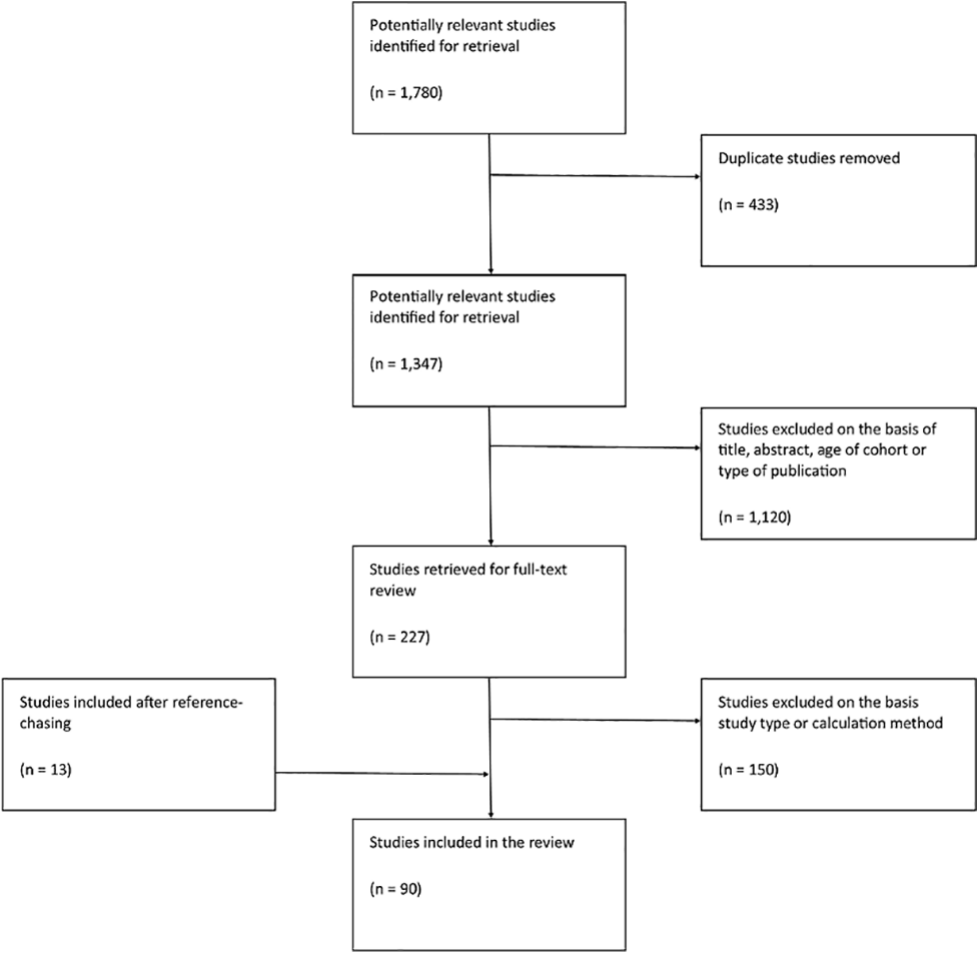
Identification of studies of measuring HRQoL in children and adolescents.

Study selection: 1,780 studies were retrieved from an online database search and were imported into an EndNote X7 library. 433 studies were removed from the list as duplicates. The remaining 1,347 studies underwent a title, abstract and type of publication review to exclude studies that did not meet the inclusion criteria. The remaining 227 studies were submitted for a full-text review. 150 studies were excluded from the full-text review as they did not use direct or indirect methods to gather primary data for HRQoL in children and adolescents, whilst an additional 13 studies were found in the list of references. In total, 90 studies were included in the review (Fig 1).

**Fig 2:**
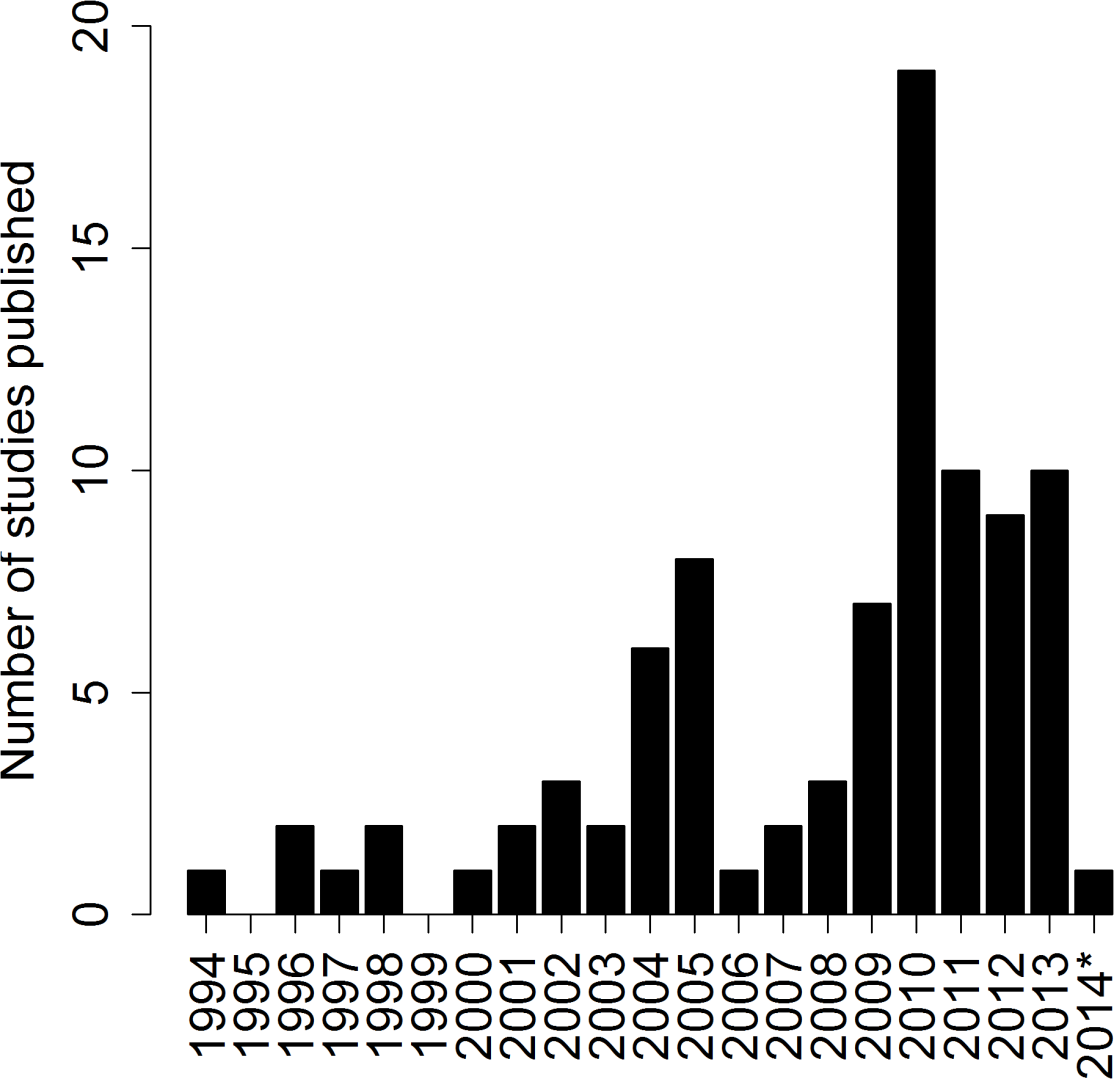
Year of publication for studies included in the review.

The earliest publication date for a study included in the review was 1994 (Fig 2). Since then the publication of measurements of health utilities in children and adolescents has steadily increased. The year with the most publications was 2010.

**Fig 3:**
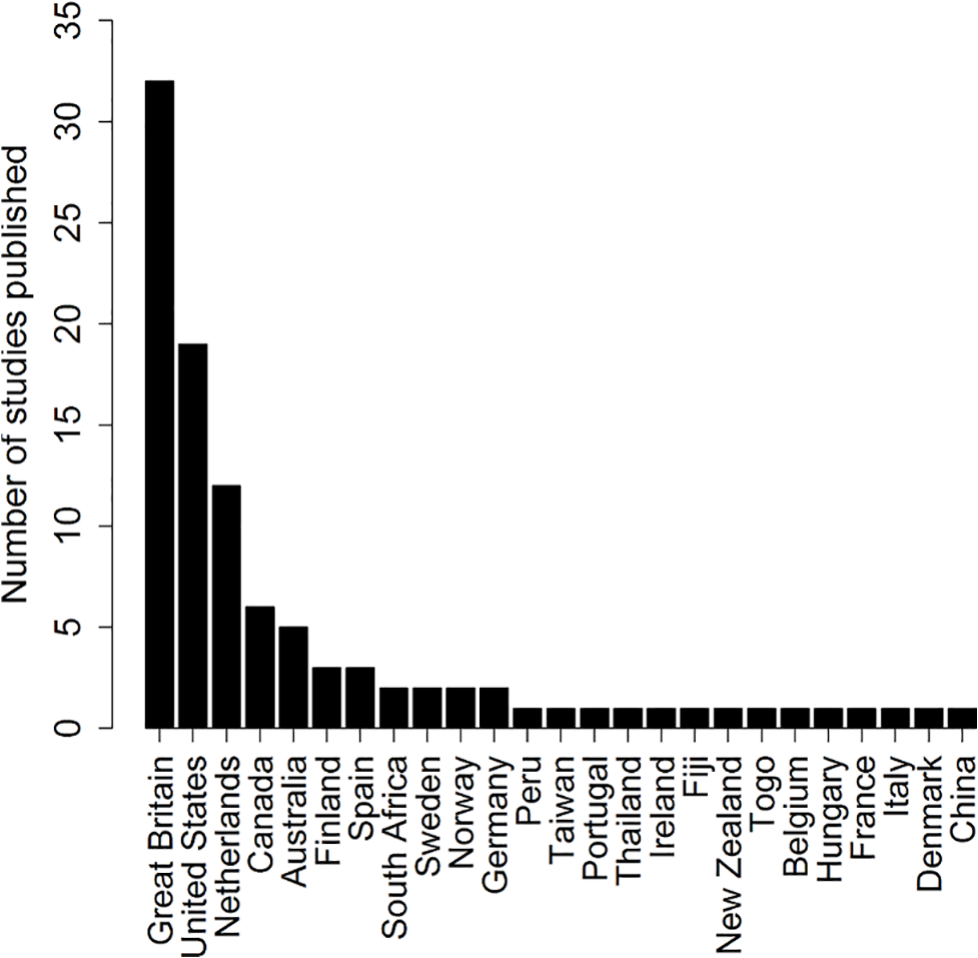
Countries featured in studies using direct or indirect calculation methods for obtaining health utilities from paediatric patients.

25 different countries were featured in the studies included in the review (Fig 3). The UK was featured the most. Three studies included multiple countries [39–41].

### Study characteristics

47 studies (52.2% of 90) were CUAs of which 21 [42–62] (44.7% of 47) were incorporated into randomised controlled trials for interventions. 23 [39, 40, 63–83] studies (25.6% of 90) were health-state utility assessments. Eight [19, 84–90] studies (8.9% of 90) were validations of calculation methods. The remaining 12 [62, 91–101] studies (13.3% of 90) were comparisons of calculation methods. 11 studies (12.2% of 90) had secondary aims of either comparing calculation methods (seven studies [62, 69, 71, 75, 77, 84, 88]) or providing health-state utility assessments (four studies [19, 41, 93, 102]).

The 90 studies used 22 unique calculation methods to gather health utilities, with the total frequency of use in all studies being 137. 7 calculation methods were disease-specific and were used 11 times (8.0% of 137) in all. The 15 generic calculation methods were used 126 times (92.0% of 137).

The EuroQol collection of indirect calculation methods was the most widely used, accounting for 38.0% of the total frequency of use (Table 2). The EQ-5D was used 41 times with its derivatives the EQ-5D-Y (used 10 times) and EQ-5D+ (a modification of the EQ-5D to include an additional dimension for cognitive functioning, used once) used separately. Direct calculation methods were also common, used 24.4% of the time. The stand-alone Visual Analogue Scale (VAS) was used 14 times, with the direct calculation methods of the SG and TTO each used nine times. The Health Utilities Index collection of indirect calculation methods was used 26 times (Table 3).

**Table 2:** Frequency of use for calculation methods found during the review.

Family of calculation method	Number of methods in family	Frequency of use
Direct Calculation	3	32
EuroQol	3	52
Health Utilities Index	2	26
Short Form	3	8
Other	11	19
	**22**	**137**

**Table 3:** Direct and indirect calculation methods to obtain health utilities from the paediatric population.

Abbreviation	Methods of obtaining utilities	Generic or disease-specific	Frequency of use
15D	15D Instrument [104]	Generic	1
ACQ	Asthma Control Questionnaire [105]	Disease specific	3
AQoL-6D	Assessment of Quality of Life 6D [106]	Generic	1
CAVE	Escala de calidad de vida del niño con epilepsia [107]	Disease specific	1
CHU-9D	Child Health Utility 9D [108]	Generic	3
EQ-5D	EuroQol 5D [109]	Generic	41
EQ-5D+	Expanded EuroQol 5D	Disease specific	1
EQ-5D-Y	EuroQol 5D Youth Version [110]	Generic	10
HALex	Health and Activities Limitation Index [111]	Generic	1
HUI-2	Health Utilities Index 2 [112]	Generic	10
HUI-3	Health Utilities Index 3 [112]	Generic	16
Mini AQLQ	Mini Asthma Quality of Life Questionnaire [113]	Disease specific	2
PAHOM	Pediatric Asthma Health Outcome Measure [19]	Disease specific	2
PAQLQ	Paediatric Asthma Quality of Life Questionnaire [114]	Disease specific	1
QLQ-C30	EORTC Quality of Life Questionnaire-Core 30 [115]	Disease specific	1
QWB	Quality of Well Being [116]	Generic	3
SF-12	Short Form 12 [117]	Generic	2
SF-36	Short Form 36 [118]	Generic	4
SF-6D	Short Form 6D [119]	Generic	2
SG	Standard Gamble [120]	Generic	9
TTO	Time Trade Off [121]	Generic	9
VAS	Visual Analogue Scale [122]	Generic	14

11 studies did not specify the age range of all participants. Four of these studies stated the mean age of participants; one study used a hypothetical cohort of child and adolescent patients but did not specify any demographic details of this hypothetical cohort; three did not give any details of the ages at all but the title and/or study details refer to child and adolescent patients; the three remaining studies indicated in aggregated results tables that some children and adolescents participated without elaboration of demographic details.

The number of participants varied from small studies of six children and adolescents [103] to studies sampling from large national databases of patients that included 84,443 patients of all ages [65] in their evaluation.

35 studies gathered health utilities exclusively from child and adolescent patients. 48 studies administered the calculation methods to adults whilst the remaining seven studies did not specify the age range of patients or did not present enough detail about the age range to determine the overall age of the cohort. 10 studies did not specify how the calculation methods were completed.

### Analysis of the use of different calculation methods

#### Measurement by proxy

54 studies administered calculation methods directly to children and adolescents in line with previous recommendations that they are able to evaluate their own health states [8–11], although 22 of these also used at least one method of proxy completion for at least one of the calculation methods. Of these 22 studies, 16 used parental proxies; four used clinician proxies; and three used caregiver proxies.

26 studies used proxies exclusively, with 17 using parental proxies, six using clinician proxies and five other proxies. One study used a combination of different proxies to obtain health utilities.

Some studies commented on the use of proxies to obtain health utilities: Cheng et al. (2000) [123] acknowledged that proxy reporting may overestimate health utility gains for cochlear implants; Chiou et al. (2005) [19] discussed issues around the use of parental proxies in their study, stating that parental preference for health may be different from child preferences; Jelsma & Ramma (2010) [97] recommended the use of self-reporting rather than proxy-reporting, acknowledging the potential issues with proxy-reporting; Oostenbrink et al. (2002) [100] stated that health utilities for CUAs should be measured from patients rather than proxies, as proxies may have difficulty evaluating the impact of conditions on dimensions of health such as pain and emotion; Tilford et al. (2005) [79] called for more research to be conducted on calculation methods for young child when discussing the issues surrounding the use of proxies; Tilford et al. (2012) [102] cite the use of proxies as a limitation in their study; Wasserman et al. (2005) [82] acknowledged a potential discrepancy between patient- and proxy-reported health utilities in their study.

However, several other studies argued that proxy-reporting was appropriate: Bichey et al. (2002) [124] said that clinician-proxy was suitable due to the clinicians’ familiarity with each case; Bodden et al. (2008) [42] referred to previous studies that used EQ-5D through proxies; Chadha et al. (2010) [93] stated that their results showed no difference between self- and proxy-reported utilities; Friedman et al. (2004) [64] claimed that parental-proxy is consistent in evaluating HRQoL for children with atopic dermatitis; Gerald et al. (2012) [88] claimed that clinician-proxy reporting of health utilities is the gold standard; Hollman et al. (2013) [67] refered to previous studies to justify their use of proxy-reporting; Matza et al. (2005) [71] claimed that SG methods through parental-proxies are a suitable method for obtaining health utilities from children; Petrou & Kupek (2009) [73] claimed that there is no consistent evidence that parental- or caregiver-proxies either over-estimate or under-estimate health utilities for their children; Poley et al. (2001) [125] cite previous studies to support the use of proxies. van Litsenburg et al. (2013) stated that the HUI-3 calculation method is a parental-proxy method by design [81].

#### Use of child- or adolescent-specific calculation methods

Six calculation methods found in this review were designed specifically for use in the child and/or adolescent population (Table 4). The number of health dimensions included ranges from three to nine. Three methods are disease-specific with two focusing on asthma and one focusing on epilepsy. The remaining three methods are generic systems.

**Table 4:** List of child- and/or adolescent-specific calculation methods used.

Abbreviation	Name of calculation method	Age range and mode of completion	Dimensions of health	Studies found using this method
AQoL-6D	Assessment of quality of life (adolescent version)	15–17 years, Self-completion	Independent living, Relationship, Mental health, Coping, Pain, Senses	[68]
CAVE	Escala de calidad de vida del niño con epilepsia	< 17 years, Self-completion, but proxy-completion for younger children	Behaviour, School compliance, Learning, Autonomy, Social relations, Frequency of seizures, Intensity of seizures, Parents opinions	[126]
CHU-9D	Child health utility, 9 dimensions	7–17 years, Self-completion, but proxy-completion for younger children	Worried, Sad, Pain, Tired, Annoyed, School work, Sleep, Daily routine, Joining with activities	[89, 90, 92]
EQ-5D-Y	EuroQol 5 dimensions, youth version	8–15 years, Self-completion	Mobility, Self-care, Usual activities, Pain or discomfort, Worried, sad or unhappy	[63, 78, 80, 83, 86, 92, 94, 96, 97, 99]
PAQLQ	Paediatric asthma quality of life questionnaire	7–17 years, Self-completion	Symptoms, Activity limitations, Emotional function	[66]
PAHOM	Pediatric asthma health outcome measure	7–12 years, Self-completion	Symptoms, Emotion, Activity	[19, 88]

Some studies discussed the short-comings of the calculation methods used. For example, Canaway et al. (2012), Oluboyede et al. (2011) and Wu et al. (2010) all discussed the lack of an appropriate tariff for the EQ-5D-Y [83, 92, 99], acknowledging that existing utilities have been taken from the adult-specific EQ-5D, finally stating that the current EQ-5D-Y is not yet complete without the child-focused tariff. Thorrington et al. (2014) also commented on the lack of a child-specific tariff for the EQ-5D-Y [78]. It has previously been noted by Kromm et al. (2012) [14] that slow progress is being made in developing age-specific utility weights.

Many other studies opted to administer calculation methods designed for a wide range of ages, such as the HUI-2 or the HUI-3. In addition, the EQ-5D system (originally designed for use in adults) was used 41 times, with the child-specific EQ-5D-Y version used only 10 times. Few studies adopting this approach discussed the suitability of their methods by evaluating the number of missing values for each returned calculation method. Hollmann et al. (2013) [67], Jelsma (2010) [96], Radford et al. (2013) [53], Thorrington et al. (2014) [78] Tilford et al. (2012) [102] and Wyatt et al. (2012) [62] all present data for missing or incomplete responses for their respective calculation methods, but only Jelsma (2010) [96] and Thorrington et al. (2014) [78] discuss these data with respect to the age of the respondents.

## Discussion

### Summary of evidence

There is extensive variation in the methods used to estimate health utilities from children and adolescents. Issues that were raised by Kromm et al. (2012) and Griebsch et al. (2005) relating to the need for a standardised method to collect health utilities from children and adolescents are yet to be fully resolved. Though this review found 22 different calculation methods that have been used between 1994 and 2013, many adult-specific methods have been used with children and adolescents without justification. Although several child- and adolescent-specific methods are currently in development, some existing adult-specific systems have been modified in order to fill the current gap.

#### Current child- and adolescent-specific calculation methods

This review found six calculation methods designed for use in children and adolescents of which the most frequently used was the EQ-5D-Y, used 10 times. Another 16 methods either designed for a wide range of ages or designed specifically for use in adults but applied to younger patients. Development and use of child- and adolescent-specific methods is steadily increasing, though several issues of suitability still surround these methods. For example, this review found that the EQ-5D-Y has been used ten times even though the EQ-5D-Y does not differentiate between adult and child or adolescent preferences for health. Several authors acknowledge this discrepancy with some calling for further research and development of child- and adolescent-specific calculation methods. At the time of writing, EuroQol has not explored child-specific utility weights that use children’s preference for health states for use in the EQ-5D-Y [110].

#### Use of proxy respondents

Justification for the use of proxy respondents was mixed, and there is no consensus for the advisability of proxy-reporting in obtaining health utilities from children and adolescents. Several studies stated that proxy-reporting may differ from self-reporting in their studies, but others claimed that their use of proxy-reporting was justified by citing previous CUAs or health utility measurements. Some studies in this review did not discuss the use of proxy-reporting vs. self-reporting and how their results may have been influenced by proxy reporting from different sources.

The use of proxies has been justified because of lack of verbal capacity of the children being evaluated [17]. Nevertheless responses should be elicited directly from those children being evaluated when verbal capacity is not a barrier [32].

#### Using multiple calculation methods and respondents

Only four studies compared self- and proxy- reported health utilities. Chadha et al. 2010 [93] found no difference between utilities. Gerald et al. (2012) [88] reported that PAHOM scores for parental proxies were significantly lower than self-reported scores from children. Jelsma & Ramma (2010) [97] found agreement with the EQ-5D-Y scores. Lock et al. (2010) [47] presented the mean and range of estimated utilities but did not perform a statistical test to verify that self-reported scores were different to proxy-reported scores.

#### Missing data

Discussions of missing data are essential in any study. In the case of the EQ-5D, a missing response to any of the five dimensions of health means that the response cannot be converted into a health utility. Analysis of missing responses would be helpful in deducing which aspects of measuring HRQoL in children and adolescents are particularly difficult and in developing new systems to minimise missing data in responses.

#### Reliance on adult-specific calculation methods

Perhaps because the EQ-5D-Y still needs an appropriate tariff for children and adolescents, some authors continue to use an adult-specific method for children and adolescents in preference to a method under development for the appropriate age group. The first use of the EQ-5D-Y in this review was in 2009 [94], and since then 18 studies have used the standard EQ-5D system in children and adolescents or patients outside of the appropriate age range for the system [41, 45, 48, 51–56, 60, 61, 67, 70, 73, 84, 91, 127, 128].

### Limitations of this review

This review only concerned published literature, which may be a source of bias as the gray literature was not considered. However, Griebsch et al. (2005) [31] argued that by not including unpublished works, they avoided reducing the overall quality of studies included in their review.

It was the decision of the authors that focused the qualitative assessment on the use and justification of different calculation methods to measure HRQoL in children and adolescents. There are several other ways to assess the quality of a CUA, notably the PQAQ [35] and the checklist for economic analysis outlined by Drummond et al. (2005) [129]. However, we have not sought to assess the quality of each CUA in the review but instead to evaluate the use of each direct or indirect calculation method in addition to understanding the justification for different methods of eliciting health utilities from children and adolescents.

## Conclusions

Many authors examining child and adolescent HRQoL have relied on tools developed exclusively for adults. Further development of child- and adolescent-specific calculation methods is required to ensure that CUAs using health utilities of children and adolescents are valid, without relying on the assumption that adults, children and adolescents all have the same health preferences.

Previous studies measuring HRQoL in children and adolescents have relied on proxy respondents without sufficient justification for their use. There is considerable debate in the literature about whether proxies can be used (and if so, which proxies). No clear consensus was found in the literature from this.

Several calculation methods are in development that will facilitate the measurement of QALYs in children. These systems are needed by health economists as the application of adult-specific systems is of questionable validity. Adults, children and adolescents measure HRQoL, perceive and value health differently, so the assumption that adult-specific health utilities are valid in adolescents or young children is potentially misleading.

Measuring children’s health states is extremely challenging and requires a suitable instrument for the estimation of paediatric health utilities that NICE can recommend for use to ensure the validity of future child- and adolescent-focused CUAs.

## Supporting Information

S1 PRISMA Checklist (DOCX)

S1 PRISMA Flowchart (DOCX)
