# Supplementary material for: Measuring Health Utilities in Children and Adolescents: A Systematic Review of the Literature
Source: PLoS One. 2015 Aug 14;10(8):e0135672. doi: 10.1371/journal.pone.0135672 (PMC4537138; doi:10.1371/journal.pone.0135672)
Supplement: S1 PRISMA Flowchart — (DOCX) [file pone.0135672.s002.docx]

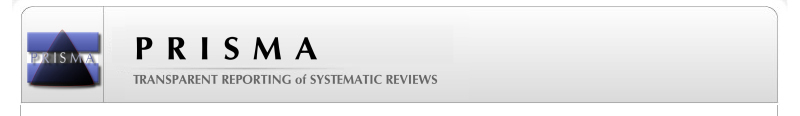
**PRISMA 2009 Flow Diagram**

Studies included in qualitative synthesis
(n = 90 )

Full-text articles excluded, with reasons
(n = 150 )

Full-text articles assessed for eligibility
(n = 240 )

Records excluded
(n = 1,120)

Records screened
(n = 1,360 )

Records after duplicates removed
(n = 433 )

Additional records identified through other sources
(n = 13)

## Identification

## Eligibility

## Included

## Screening

Records identified through database searching
(n = 1,780 )
